# Dynamic analysis of the relationship between systemic lupus erythematosus disease activity and psychosocial support

**DOI:** 10.3389/fpsyg.2024.1433868

**Published:** 2024-08-14

**Authors:** Miao Lu, Min Liu, Kuijun Zhan, Yutong Chen, Xide Liu

**Affiliations:** ^1^Second Clinical College, Zhejiang Chinese Medicine University, Hangzhou, Zhejiang, China; ^2^Department of Traditional Chinese Medicine, Hangzhou Red Cross Hospital, Zhejiang Province, Hangzhou, Zhejiang, China

**Keywords:** systemic lupus erythematosus, disease activity, psychosocial support systems, retrospective studies, longitudinal studies, social support

## Abstract

**Background:**

Systemic lupus erythematosus (SLE) is a multi-system autoimmune disease that significantly affects both the physical and mental health of patients. Psychosocial support systems play a crucial role in managing chronic diseases, yet their specific impact on the disease activity of SLE patients remains unclear. This study aims to explore the dynamic relationship between disease activity in SLE patients and various types of psychosocial support systems.

**Methods:**

We conducted a retrospective longitudinal observational study, including 150 SLE patients who received treatment at our hospital from January 2022 to January 2023. Emotional support, tangible support, social interaction support, and informational support were assessed using the revised Social Support Rating Scale. Disease activity was quantified using the European Consensus Lupus Activity Measurement. The relationship between psychosocial support and disease activity was analyzed using Spearman’s rank correlation coefficient and multiple linear regression models, with Bootstrap resampling employed to test the robustness of the results.

**Results:**

We found a significant negative correlation between psychosocial support and SLE disease activity, with emotional support, social interaction support, and informational support showing stronger negative correlations. Multiple regression analysis revealed that the inhibitory effects of emotional support, social interaction support, and informational support on disease activity increased over time. Although the impact of tangible support was not statistically significant, it gradually became more apparent over time.

**Conclusion:**

Our findings indicate a significant negative correlation between psychosocial support and SLE disease activity, particularly with emotional support, social interaction support, and informational support. Over time, the impact of tangible support also becomes evident. These findings provide important references for the comprehensive treatment and management of SLE patients. However, due to the observational nature of the study, the causality of this relationship requires further exploration.

## Introduction

Systemic lupus erythematosus (SLE) is a complex multi-system autoimmune disease characterized by widespread immune dysregulation and diverse clinical manifestations (Lu et al., 2016; Qi et al., 2019; Tsai et al., 2020). The disease activity of SLE is extensive and variable, encompassing arthritis, skin lesions, renal involvement, and even neurological manifestations. SLE poses significant threats to patients’ physical health and severely impairs their psychological and social functioning (Phuti et al., 2018; Ghosh et al., 2020; Shi et al., 2021). In recent years, the importance of psychosocial support in managing chronic diseases has garnered increasing attention. Psychosocial support, obtained through social networks and interactions, is a multidimensional support system comprising emotional support, tangible support, social interaction support, and informational support (Mancuso et al., 2011; Mazzoni and Cicognani, 2014; Paladino et al., 2019; Hurtado-de-Mendoza et al., 2022). Emotional support refers to care, understanding, and emotional comfort, while tangible support involves practical assistance such as money or goods. Social interaction support provides opportunities for participation in recreational or leisure activities, and informational support includes advice, information, and guidance. These forms of support may positively impact patients’ health by alleviating psychological stress, improving health behaviors, and enhancing patients’ sense of control over their disease (Chang et al., 2021; Piga and Arnaud, 2021; Ra et al., 2021). Although existing research has demonstrated the positive impact of psychosocial support on the health status of patients with chronic diseases, there is still insufficient research on how psychosocial support specifically affects disease activity in SLE patients, as well as the mechanisms and extent of its effects. Current studies mainly focus on the relationship between social support and mental health, with limited exploration of how social support influences the biological and clinical manifestations of SLE (Dobkin et al., 2002; Pan et al., 2019; Chen et al., 2022; Williams et al., 2023). Moreover, different types of psychosocial support may have varying effects on SLE disease activity. For example, emotional and informational support might significantly reduce disease activity by improving psychological well-being and increasing disease-related knowledge, whereas the short-term effects of tangible support may be less pronounced compared to other types of support. However, the impact of tangible support may become more evident over time (Mazzoni and Cicognani, 2016; Chaigne et al., 2017; Barraclough et al., 2024; Wang et al., 2024). Based on this, the present study aims to conduct a retrospective longitudinal observational study to explore the relationship between different types of psychosocial support (emotional support, tangible support, social interaction support, and informational support) and SLE disease activity. Additionally, we will analyze the temporal trends of these relationships to provide clinical practitioners and public health policymakers with in-depth insights. This will aid in better understanding and utilizing social support networks, ultimately optimizing treatment and management strategies for SLE patients.

## Methods

This study employs a retrospective longitudinal observational method, systematically including SLE patients who received treatment at our hospital from January 2022 to January 2023 and signed informed consent forms. Detailed clinical data were collected from these patients at the start of the study and subsequently at 4, 8, and 12 months. The aim is to deeply explore the association between different types of psychosocial support and SLE disease activity, and to further investigate the dynamic characteristics of this relationship over time. The ultimate goal is to provide valuable references and guidance for the clinical treatment and management of SLE.

### Study participants

#### Inclusion criteria

(1) Adults aged 18–65 years. (2) Clinically diagnosed with SLE according to the American College of Rheumatology (ACR) criteria (Yu et al., 2014). (3) At least one disease activity assessment recorded by a rheumatologist within 6 months prior to the study. (4) Ability to understand the study requirements and voluntarily sign the informed consent form. (5) Ability to provide personal psychosocial support information through interviews or questionnaires.

#### Exclusion criteria

(1) History of severe cognitive impairment or mental illness that could affect the accuracy of the information provided or understanding of the study requirements. (2) Major SLE-related complications such as severe heart disease or renal failure, which may require special treatment or monitoring during the study. (3) Pregnant or breastfeeding women. (4) Patients who have not received stable standard anti-inflammatory or immunomodulatory treatment within the 12 months prior to the study. (5) Currently participating in other clinical trials, to avoid the influence of interventions or drug interactions on the study results. (6) Recent use of medications that may affect psychological status (e.g., antidepressants, sedatives).

### Data collection

#### Baseline data collection

At the time of enrollment, all patients were required to complete a detailed intake questionnaire, which included the following sections:

1. Demographic Information: Age, gender, ethnicity, marital status, education level, and occupation.
2. Disease History and Treatment Information: Detailed record of the duration of SLE and current treatment methods.
3. Health Behaviors: Smoking and drinking habits, and frequency of physical activity.
4. Psychological Health Status: Assessed using widely recognized psychological health scales such as the Center for Epidemiologic Studies Depression Scale (CES-D) and the Generalized Anxiety Disorder 7-item scale (GAD-7) (Byrd-Bredbenner et al., 2020; Uchida et al., 2023).

#### Psychosocial support assessment

Psychosocial support was assessed using the revised Social Support Rating Scale (MOS-SSS-C) (Yu et al., 2004). The MOS-SSS-C is specifically designed for chronic disease patients to evaluate multidimensional support obtained from social networks. As the gold standard for psychosocial assessment, this tool is crucial for understanding patients’ social interactions and support systems. The scale includes emotional support (e.g., caring, affection, and emotional understanding), tangible support (e.g., financial assistance, borrowing items, or other practical help), social interaction support (e.g., opportunities for participation in entertainment or leisure activities), and informational support (e.g., advice, information, guidance, or feedback to help solve problems or make decisions). This scale has been validated for high reliability and validity, ensuring the accuracy and reliability of the assessment results (Merino-Soto et al., 2023; Zeng et al., 2024).

#### Disease activity assessment

The assessment of SLE disease activity was conducted using the European Consensus Lupus Activity Measurement (ECLAM) (Zhang et al., 2021; Floris et al., 2022). ECLAM is a comprehensive scoring system designed to quantify SLE disease activity by integrating multiple clinical and laboratory parameters to calculate a total score. This system covers a range of clinical symptoms and laboratory indicators, including arthritis, skin lesions, mucosal ulcers, alopecia, and thrombocytopenia, thus providing a comprehensive reflection of SLE activity. The ECLAM score ranges from 0 to 10, with higher scores indicating greater disease activity.

### Follow-up

During the study, participants were regularly followed up to update their disease status and any changes in psychosocial support. This included a follow-up questionnaire survey conducted via telephone or face-to-face interviews at 1, 4, 8, and 12 months, covering changes in psychosocial support and disease activity assessment.

### Data analysis methods

All statistical analyses in this study were performed using SPSS and R software to ensure rigorous and high-standard data processing. First, descriptive statistics were used to detail the demographic characteristics and disease-related information of the participants. The Spearman rank correlation coefficient was employed to evaluate the correlation between psychosocial support scores and SLE disease activity scores at the beginning of the study. Multiple linear regression models were used to analyze the independent effects of psychosocial support on SLE disease activity, controlling for key confounding factors such as age, gender, and disease history, to ensure the accuracy and reliability of the results. Sensitivity analysis was conducted using the Bootstrap resampling method to further explore the dynamic relationship and potential mechanisms between psychosocial support and disease activity. Additionally, by comparing regression coefficients at 1, 4, 8, and 12 months from the start of the study, we further investigated the dynamic characteristics of this relationship over time.

### Ethical considerations

This study strictly adhered to ethical guidelines and received approval from the Ethics Committee of our hospital. All participants signed informed consent forms, understanding that their participation was voluntary and that they could withdraw at any time. Personal information of the participants was anonymized and accessible only to authorized researchers. As a cross-sectional observational study, there was no intervention in the patients’ conditions. The final results of the study will be publicly released to ensure transparency and public awareness. These measures ensured the protection of participants’ rights while maintaining the scientific and ethical integrity of the study.

## Results

### Basic characteristics of the study population

A total of 150 SLE patients were included in this study. Among them, 22 were male (14.7%) and 128 were female (85.3%), with an average age of 35.6 ± 6.1 years. In terms of marital status, 54.9% were married, 35.1% were unmarried, and the remaining 10.0% were divorced. Regarding education level, 35.3% of the patients had a college degree or higher, while 64.7% had a high school education or less. Concerning employment status, 79.3% of the participants were employed full-time, 8.2% part-time, and 12.5% were currently unemployed. Disease history revealed an average disease duration of 7.1 ± 8.6 years. In terms of current treatment, the majority of patients (80.8%) were receiving immunosuppressants, 71.4% were using non-steroidal anti-inflammatory drugs (NSAIDs), and 37.6% were being treated with biologics. Regarding health behaviors, 61.7% of the patients never smoked, 26.9% never drank alcohol, and only 22.1% engaged in physical activity three times or more per week. In psychological health assessments, the average score on the CES-D scale was 16.2 ± 5.9, indicating some degree of depressive symptoms, while the average score on the GAD-7 scale was 10.6 ± 4.7, reflecting moderate anxiety symptoms (Table 1).

**Table 1:** Basic characteristics of the study population.

Characteristic	Value
Total participants (*n*)	150
Average age (*M* ± SD)	35.6 ± 6.1
Gender (female/male)	128/22
Marital status (married/single/divorced)	54.9/35.1/10.0%
Education (college and above/high school or below)	35.3/64.7%
Employment (full-time/part-time/unemployed)	79.3/8.2/12.5%
Disease duration (years)	7.1±8.6
Current treatment methods (immunosuppressant/NSAIDs/biologics)	80.8/71.4/37.6%
Smoking habit (non-smoker/others)	61.7/38.3%
Drinking habit (non-drinker/others)	26.9/73.1%
Weekly physical activity (3 times or more/less than 3 times)	22.1/77.9%
CES-D score (depression)	16.2 ± 5.9
GAD-7 score (anxiety)	10.6 ± 4.7

### Psychosocial support assessment and disease activity score

The MOS-SSS-C scale assessment shows that all participants exhibited varying levels of the four types of psychosocial support. Specifically, at the beginning of the study (Month 1), social interaction support had the highest average score (16.00 ± 5.93), while emotional support had the lowest average score (14.72 ± 5.72). Tangible support had a lower and more scattered distribution (15.71 ± 7.10), whereas informational support had a higher and more concentrated distribution (15.85 ± 4.60). Over time, all types of support showed an increasing trend, with informational support showing the most significant growth. All patients were assessed for SLE disease activity according to the ECLAM standards, revealing that SLE disease activity gradually decreased from a higher level in Month 1 to a lower level in Month 12. This trend partly reflects the increase in psychosocial support as disease activity decreases (Table 2).

**Table 2:** Psychosocial support assessment and disease activity score.

Type of support/SLE disease activity	Mean ± standard deviation			
	January	April	August	December
Emotional support	14.72 ± 5.72	16.24 ± 5.01	17.52 ± 4.56	19.14 ± 4.15
Tangible support	15.71 ± 7.10	16.18 ± 7.59	18.36 ± 5.23	19.75 ± 4.08
Social interaction support	16.00 ± 5.93	16.44 ± 5.37	17.62 ± 4.79	18.84 ± 4.45
Informational support	15.85 ± 4.60	16.65 ± 4.13	18.67 ± 3.34	20.48 ± 3.12
SLE disease activity	6.49 ± 1.03	6.02 ± 1.01	5.27 ± 0.88	4.14 ± 0.63

### Main analysis results

#### Correlation analysis

**Figure 1:**
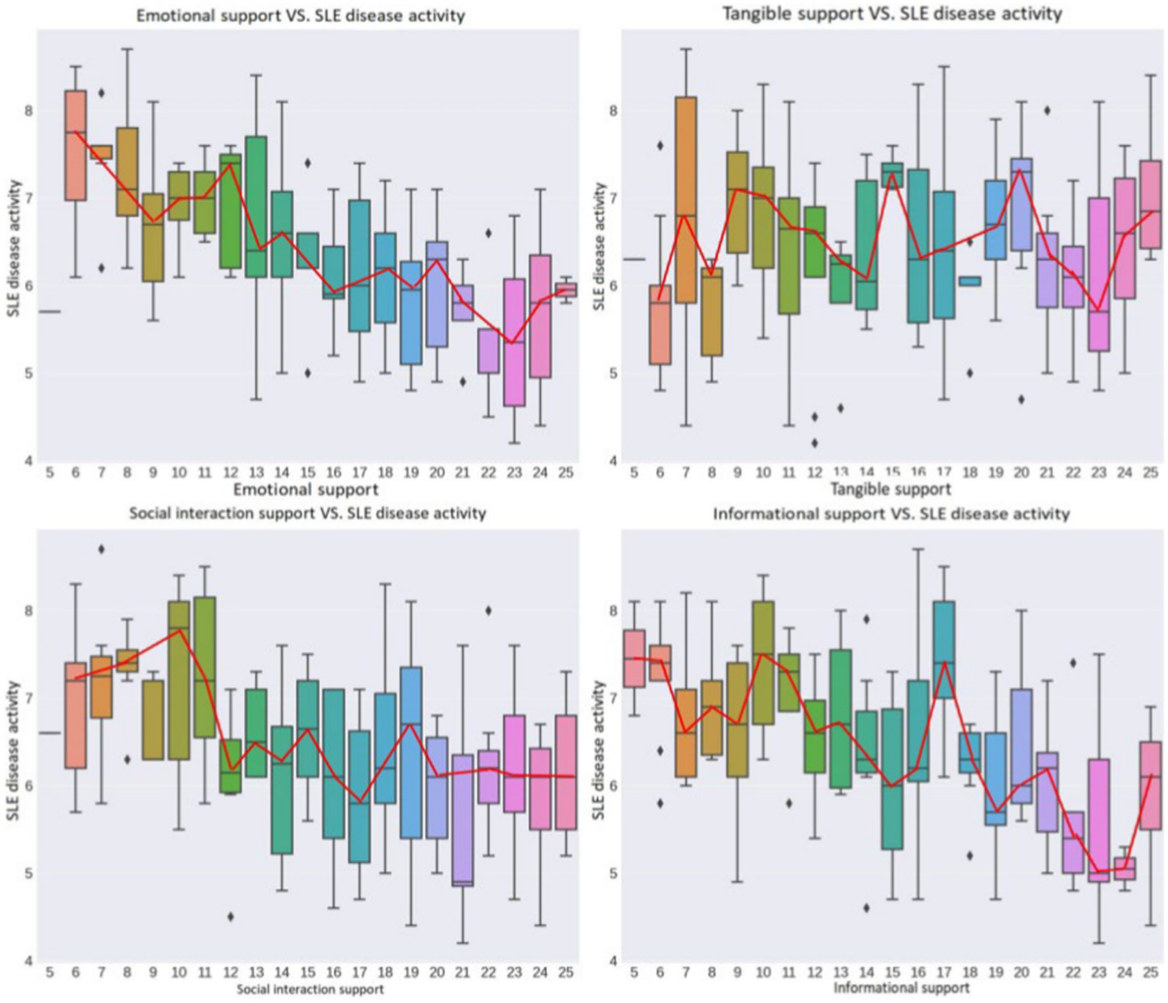
Correlation analysis of different types of psychosocial support and SLE disease activity. Higher scores in emotional support, social interaction support, and informational support are generally associated with lower disease activity, reflected in the downward trend of the box plot. The relationship between tangible support and SLE activity is less clear, with a more uniform distribution, suggesting that tangible support may have a minimal impact on SLE activity.

In this study, we used the Spearman rank correlation coefficient to analyze the relationship between different types of psychosocial support and SLE disease activity. The results showed a significant negative correlation between emotional support and SLE disease activity (correlation coefficient −0.592), indicating that increased emotional support is associated with decreased disease activity. In contrast, tangible support had a very low correlation with disease activity (correlation coefficient 0.067), suggesting no significant association between the two. Additionally, social interaction support (correlation coefficient −0.395) and informational support (correlation coefficient −0.460) both showed negative correlations, indicating that increases in these types of support may help reduce disease activity (Figure 1).

### Multivariable regression analysis

**Figure 2:**
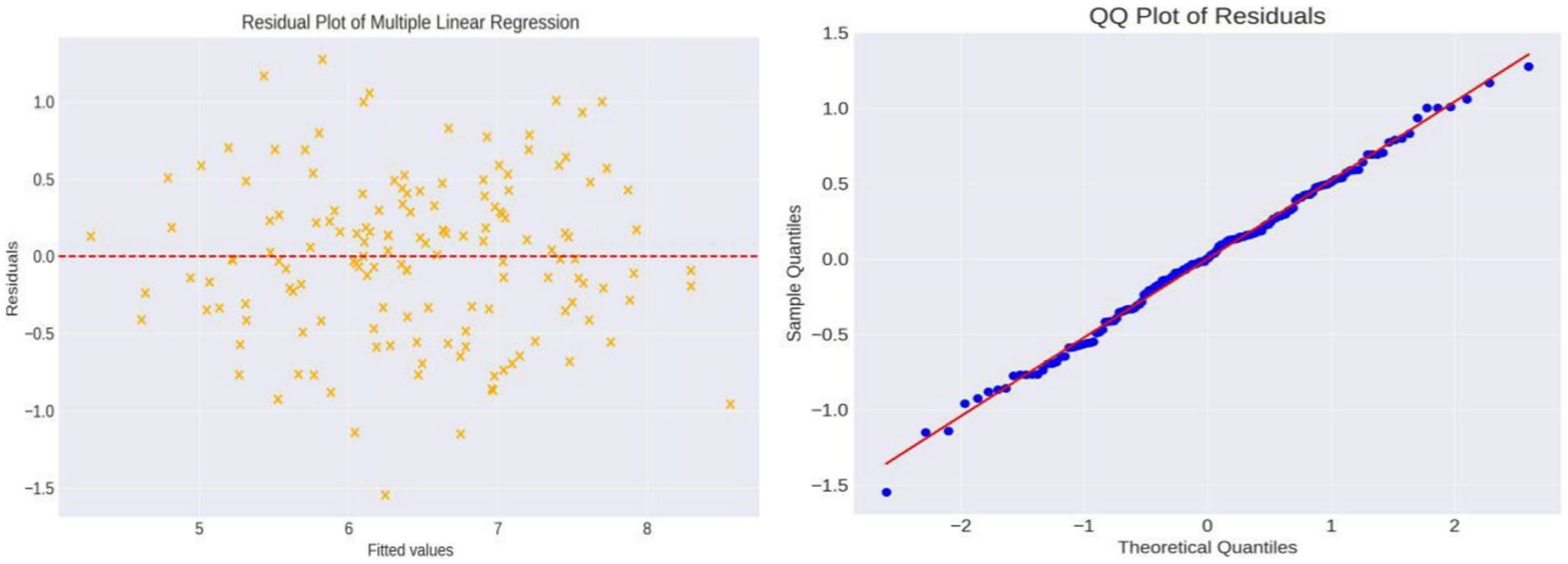
Residual plot and QQ plot of the multivariable regression analysis. The residual plot shows the differences between actual and predicted values and their relationship with fitted values. Observing the residual distribution, we find that data points are randomly distributed around the zero line, with no obvious systematic bias, indicating a good model fit. The QQ plot shows that most data points closely follow the reference line, suggesting that the residuals are approximately normally distributed, which is a good sign for the reliability of the statistical inference in this linear regression model.

After controlling for potential confounding factors such as age, gender, and disease duration, we conducted multiple linear regression analysis. The adjusted *R*^2^ value was 0.726, indicating that the model could explain approximately 72.6% of the variability in SLE disease activity, demonstrating good model fit. The regression coefficients for emotional support, social interaction support, and informational support were −0.1108, −0.0604, and −0.0806, respectively. This means that for each unit increase in these supports, SLE disease activity decreases by an average of 0.1108, 0.0604, and 0.0806 units, respectively, all of which are statistically significant (*p* < 0.001). However, the regression coefficient for tangible support was only −0.0031, indicating its minimal and statistically insignificant impact on SLE disease activity (*p* = 0.668). The overall model’s *F*-value was 99.87 with a *p*-value close to 0, well below the 0.05 significance level, strongly suggesting that at least one explanatory variable in the model significantly affects SLE disease activity (Figure 2).

### Sensitivity analysis

**Figure 3:**
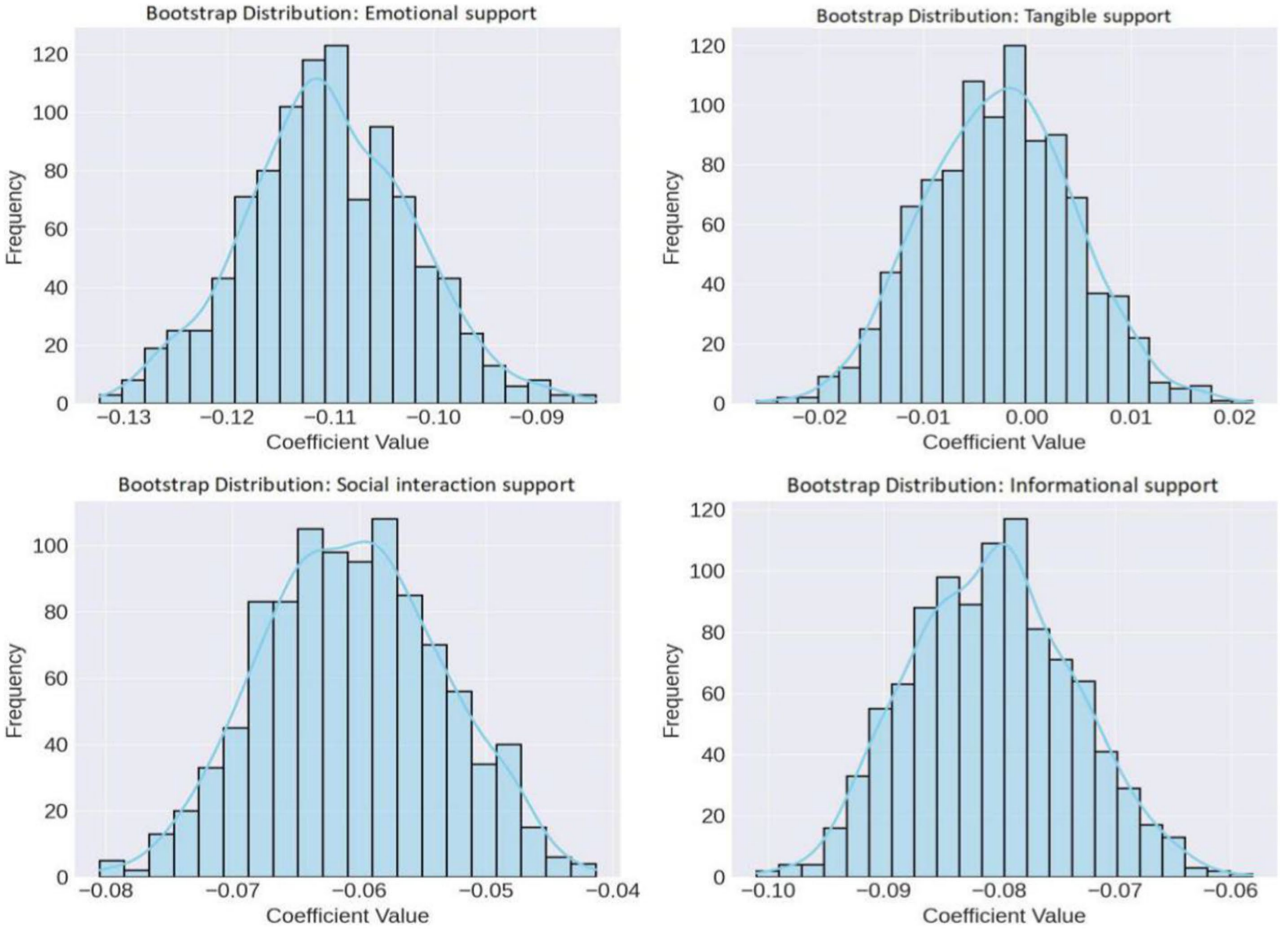
Sensitivity distribution of Bootstrap regression coefficients. The regression coefficients for emotional support, social interaction support, and informational support are relatively stable, showing strong negative correlations and low uncertainty, whereas the impact of tangible support scores is less evident and more variable.

We used the Bootstrap resampling method to evaluate the impact of different types of psychosocial support on SLE disease activity, aiming to investigate the variability and stability of the model parameters. After 1,000 Bootstrap re-samplings, we found that the regression coefficients for emotional support, social interaction support, and informational support were centered around −0.110, −0.0606, and −0.0806, respectively, indicating strong negative correlations and low uncertainty. In contrast, the regression coefficient for tangible support fluctuated around −0.0027, with a larger standard deviation, reflecting higher uncertainty (Figure 3).

### Trend analysis of psychosocial support regression coefficients

**Figure 4:**
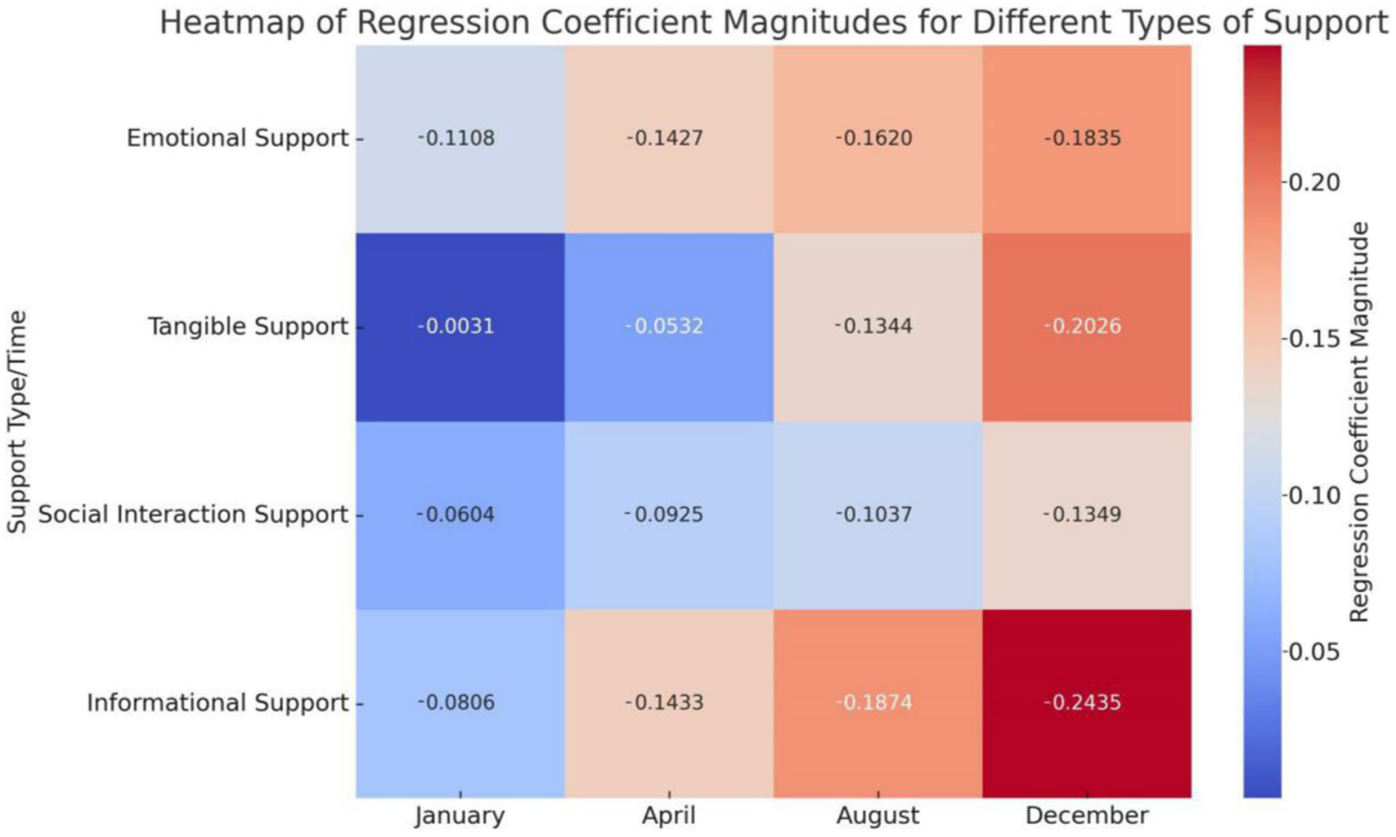
Heatmap of regression coefficients for four types of psychosocial support. The heatmap shows the regression coefficients for four types of psychosocial support at 1, 4, 8, and 12 months. Color changes represent the magnitude of the regression coefficients: the closer the color is to red, the larger the regression coefficient; the closer the color is to blue, the smaller the regression coefficient. “−” only indicates a negative correlation and does not represent the magnitude.

Based on the study’s initial month, we conducted systematic multiple linear regression analyses at key time points (April, August, and December). The results showed that the regression coefficient for emotional support exhibited a stable growth trend, rising from 0.1108 in January to 0.1835 in December, showing an orderly increase throughout the year. Similarly, social interaction support and informational support displayed a consistent upward trend, increasing from lower levels at the beginning of the year to higher levels by year-end. In contrast, the regression coefficient for tangible support was very low in January (0.0031) but significantly increased to 0.2026 by December, showing a notable growth trend (Figure 4).

In summary, the final results demonstrate that emotional support, social interaction support, and informational support are significantly negatively correlated with SLE disease activity, and these effects gradually strengthen over time. Multiple linear regression analysis confirmed these findings, showing that the impacts of emotional support, social interaction support, and informational support are statistically significant and stable. Although the initial effect of tangible support was minimal, its influence increased by the end of the study. Bootstrap resampling validated the stability of the model, indicating that the effects of emotional support, social interaction support, and informational support are relatively stable, whereas the impact of tangible support exhibits greater uncertainty.

## Discussion

SLE, as a chronic autoimmune disease, affects not only the physical health of patients but also poses challenges to their psychological and social functions (Phuti et al., 2018; Qi et al., 2019; Ghosh et al., 2020). In recent years, with the transformation of the medical model, there has been increasing recognition of the impact of psychosocial factors on disease progression (Mancuso et al., 2011; Mazzoni and Cicognani, 2014; Shi et al., 2021). In this context, our study systematically explored the relationship between psychosocial support and SLE disease activity and its dynamic changes, aiming to provide important insights for further understanding the complexity of SLE and designing more comprehensive intervention strategies.

### Correlation analysis

At the beginning of the study in January, we found a significant negative correlation between emotional support, social interaction support, and informational support and SLE disease activity. Emotional support, encompassing care, affection, and deep emotional understanding, directly addresses the emotional needs of patients, effectively alleviating their psychological stress and emotional distress. This support enhances patients’ psychological resilience and positive emotions, strengthens their coping strategies, and self-management abilities, thereby positively impacting the reduction of SLE disease activity (Natale et al., 2019; Farhat et al., 2020; Kyriakou et al., 2020). Social interaction support provides SLE patients with social opportunities, helping them maintain connections with society. This not only effectively improves patients’ social functioning and alleviates feelings of loneliness and depression but also further promotes their psychological health (Carr et al., 2011; Henry et al., 2019; Cohen and Holtzer, 2023). Informational support aids patients in better understanding their disease, enhancing their knowledge and self-management capabilities, which may lead to more effective disease management and possibly lower disease activity (Jump et al., 2005; Cui et al., 2019; Williams et al., 2023). However, although tangible support offers economic and material convenience to patients, its short-term effects on directly improving their biomedical status are limited. Consequently, its impact on enhancing patients’ psychological state or improving disease self-management behaviors is not significant in the short term (Woloshin et al., 1997; Panagioti et al., 2014a,b; Bee et al., 2018).

### Dynamic changes in correlations

In the subsequent phases of the study, we observed a trend where the inhibitory effects of emotional support, social interaction support, and informational support on SLE disease activity gradually increased over time. Specifically, the suppressive impact of emotional support significantly improved during the study period. This may be attributed to patients developing deep trust and reliance through sustained emotional care, which enables them to better regulate their emotions and face treatment more positively. Furthermore, the enhancement of social interaction support also demonstrated its long-term benefits in improving patients’ social functioning and alleviating psychological stress, indicating that ongoing social engagement helps patients better integrate into society, reduce feelings of loneliness, and positively impact their overall health status (Jump et al., 2005; Arabyat and Raisch, 2019; Wang et al., 2019; Walburn et al., 2020; Blackie et al., 2023; Wanberg and Pearson, 2024). Additionally, the increasing significance of informational support on patients’ self-management suggests that patients are progressively learning to use health information to manage their disease effectively. Although the direct impact of tangible support on SLE disease activity was minimal at the beginning of the study, its influence became more significant over time. This could be due to the long-term material assistance subtly improving patients’ quality of life and treatment adherence (Harry et al., 2019; Gao et al., 2022; Moore-Bouchard et al., 2024).

### Potential clinical applications

This study highlights the importance of providing emotional support, social interaction support, and informational support in the comprehensive management of SLE patients. Clinicians should encourage patients to establish and maintain strong social support networks by increasing emotional comfort, promoting social interaction, and providing disease-related information to effectively manage SLE disease activity. Although the initial impact of tangible support is limited, it should not be overlooked in long-term management, as economic and material assistance can gradually improve patients’ quality of life and treatment adherence. Future research should expand the sample size and include more cultural and socioeconomic factors to further validate the study’s conclusions. Additionally, exploring the specific mechanisms of different types of support in various populations will help develop more personalized and effective intervention strategies, thereby optimizing the overall health management of SLE patients.

### Limitations

Despite providing valuable insights into the roles of emotional, social, and informational support in the management of SLE, this study has several limitations. First, the limited sample size may restrict the generalizability and statistical power of the findings. Second, the observational study design allows for the observation of associations between psychosocial support and SLE disease activity, but it does not establish causality. This needs to be confirmed through more rigorous research methods, such as randomized controlled trials. Additionally, the assessment of patients’ psychosocial status was primarily based on self-reports, which may introduce subjective bias. Lastly, the study did not adequately consider the impact of cultural and socioeconomic factors on the relationship between types of support and disease activity. Future research should further explore these aspects to provide a more comprehensive understanding.

## Conclusion

Emotional support, social interaction support, and informational support exhibit a significant and progressively increasing negative correlation with SLE disease activity. In contrast, the impact of tangible support is smaller and less statistically significant in the early stages of the study, but its negative correlation gradually increases over time. However, due to the observational nature of this study, the causality of these associations needs further investigation. Understanding these causal relationships will aid in developing more personalized and effective intervention strategies, thereby optimizing the overall health management of SLE patients.

## Data availability statement

The original contributions presented in the study are publicly available. This data can be found here: https://www.scidb.cn/en/anonymous/WWpNenll.

## Ethics statement

The studies involving human participants were reviewed and approved by the Medical Ethics Committee of Hangzhou Red Cross Hospital. The studies were conducted in accordance with the local legislation and institutional requirements. The participants provided their written informed consent to participate in this study.

## Author contributions

MLu: Data curation, Formal analysis, Methodology, Resources, Writing – original draft. MLi: Formal analysis, Investigation, Writing – original draft, Visualization. KZ: Formal analysis, Investigation, Resources, Writing – original draft. YC: Investigation, Visualization, Writing – original draft. XL: Conceptualization, Data curation, Methodology, Project administration, Writing – review & editing.
